# Heme oxygenase-1 ameliorates oxidative stress-induced endothelial senescence via regulating endothelial nitric oxide synthase activation and coupling

**DOI:** 10.18632/aging.101506

**Published:** 2018-07-24

**Authors:** Wenwei Luo, Yu Wang, Hanwei Yang, Chunmei Dai, Huiling Hong, Jingyan Li, Zhiping Liu, Zhen Guo, Xinyi Chen, Ping He, Ziqing Li, Fang Li, Jianmin Jiang, Peiqing Liu, Zhuoming Li

**Affiliations:** 1 Laboratory of Pharmacology and Toxicology, School of Pharmaceutical Sciences; National and Local United Engineering Lab of Druggability and New Drugs Evaluation; Guangdong Provincial Key Laboratory of New Drug Design and Evaluation, Sun Yat-sen University, Guangzhou 510006, China; 2 Infinitus (China) Co. Ltd, Guangzhou 510663, China; 3 College of Life Science, South China Agricultural University, Guangzhou 510642, China; * Equal contribution

**Keywords:** endothelial cell, endothelial nitric oxide synthase, heme oxygenase-1, nitric oxide, senescence

## Abstract

Aim: Premature senescence of vascular endothelial cells is a leading cause of various cardiovascular diseases. Therapies targeting endothelial senescence would have important clinical implications. The present study was aimed to evaluate the potential of heme oxygenase-1 (HO-1) as a therapeutic target for endothelial senescence.

Methods and Results: Upregulation of HO-1 by Hemin or adenovirus infection reversed H_2_O_2_-induced senescence in human umbilical vein endothelial cells (HUVECs); whereas depletion of HO-1 by siRNA or HO-1 inhibitor protoporphyrin IX zinc (II) (ZnPP) triggered HUVEC senescence. Mechanistically, overexpression of HO-1 enhanced the interaction between HO-1 and endothelial nitric oxide synthase (eNOS), and promoted the interaction between eNOS and its upstream kinase Akt, thus resulting in an enhancement of eNOS phosphorylation at Ser1177 and a subsequent increase of nitric oxide (NO) production. Moreover, HO-1 induction prevented the decrease of eNOS dimer/monomer ratio stimulated by H_2_O_2_ via its antioxidant properties. Contrarily, HO-1 silencing impaired eNOS phosphorylation and accelerated eNOS uncoupling. *In vivo*, Hemin treatment alleviated senescence of endothelial cells of the aorta from spontaneously hypertensive rats, through upregulating eNOS phosphorylation at Ser1177.

Conclusions: HO-1 ameliorated endothelial senescence through enhancing eNOS activation and defending eNOS uncoupling, suggesting that HO-1 is a potential target for treating endothelial senescence.

## Introduction

Vascular endothelial cells, the inner layer of the vasculature, play a pivotal role in regulating hemostasis of the cardiovascular system, such as vascular tone, balance between thrombosis and thrombolysis, recruitment of inflammatory cells to the vasculature, platelet aggregation, etc [1]. In various pathophysiological conditions, however, endothelial cells underlying stimuli such as oxidative stress, angiotensin II, high glucose and high lipids, develop a premature senescent phenotype [2]. This “stress-induced premature senescence” of endothelial cells is characterized by cell-cycle arrest, and pro-adhesive, pro-inflammatory and pro-thrombotic changes in gene expressions [2]. Endothelial senescence results in endothelial dysfunction and promotes the progression of vascular diseases like atherosclerosis, subsequently raising the cardiovascular risk such as myocardial infarction and stroke [3–6]. Thus, therapies targeting endothelial senescence would have important clinical implications for the treatment of cardiovascular diseases [7,8].

Heme oxygenase-1 (HO-1) has been identified as the rate-limiting enzyme in the degradation of heme to release free iron, carbon monoxide (CO) and biliverdin [9]. The transcription and expression of HO-1 is regulated by the transcription factor nuclear erythroid 2-related factor 2 (Nrf2). Under cellular stress induced by a number of chemical and physical stimuli, including heavy metals, endotoxin, cytokines, heme, hypoxia, heat shock and UV irradiation, Nrf2 is disassociated with its inhibitory protein Kelch-like ECH-associated protein 1 (KEAP1) and is transported to the nucleus, finally binds to antioxidant response element (ARE) to elicit the transcription of HO-1 [10]. Besides, HO-1 can be induced by a variety of drugs and natural products, such as Hemin [11,12], salvianolic acid A [13], curcumin [14], etc. Because of its critical role in attenuating the intracellular production of reactive oxygen species (ROS) through its ability to break down the heme moiety and to generate the cytoprotective CO and bilirubin (which is converted by biliverdin), HO-1 has been considered as one of the most important endogenous protective protein against oxidative stress and organ damage [15].

A large body of evidences support that HO-1 plays a fundamental role in maintaining cardiovascular homeostasis. HO-1 induction reverses atherosclerotic lesion progression from a vulnerable plaque to a more stable phenotype [16]. HO-1 upregulation demonstrates anti-hypertensive effects [17–21]. Our previous studies have found that HO-1 induction prevents endothelial dysfunction in hypertension, through inhibiting the production and release of endothelium-derived contracting factors (EDCFs) and potentiating endothelium-dependent hyperpolarization (EDH) [20,21]. These findings provoke our interest in the cardiovascular protective effects of HO-1 and its therapeutic potential in treating various diseases associated with endothelial dysfunction. Since endothelial dysfunction is resulted from senescence of the endothelial cells, it is hypothesized that HO-1 participates in the regulation of endothelial senescence. Indeed, recent studies indicate that up-regulation of HO-1 could be detected in senescent astrocytes [22], fibroblasts [23], epidermal keratinocytes [24], and pancreatic islet cells [25]. Moreover, HO-1 activation plays an important role in maintaining activities of endothelial progenitor cells and resisting senescence in vascular smooth muscle cells induced by angiotensin II [26]. These studies suggest that HO-1 may be closely associated with cell senescence. Thus, the present study was designed to investigate the effect and the underlying mechanisms of HO-1 on endothelial senescence.

## RESULTS

### Upregulation of HO-1 by Hemin or HO-1 adenovirus ameliorated H_2_O_2_-induced endothelial senescence

**Figure 1:**
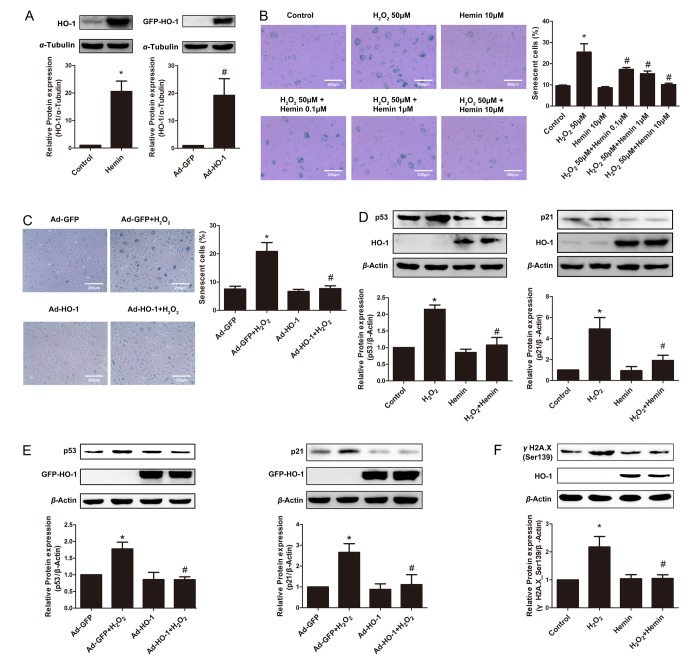
**Induction or overexpression of HO-1 reversed HUVEC senescence.** (**A**) Upregulation of HO-1 protein expression in HUVEC induced by Hemin or transfected with HO-1 recombinant adenovirus (HO-1, 32kDa; GFP-HO-1, 59kDa). ^*^P < 0.05 vs. Control; ^#^P < 0.05 vs. Ad-GFP. n = 5. (**B**) HUVEC were stained for SA-β-galactosidase treated with H_2_O_2_ (50 μM, 1 h) or/and Hemin (0.1-10 μM). Senescent cells appeared blue (200 × magnification). ^*^P < 0.05 vs. Control; ^#^P < 0.05 vs. H_2_O_2_. n = 5. (**C**) SA-β-gal staining of HUVEC treated with H_2_O_2_ (50 μM, 1 h) or/and HO-1 recombinant adenovirus (200 × magnification). ^*^P < 0.05 vs. Control; ^#^P < 0.05 vs. Ad-GFP+H_2_O_2_. n = 5. (**D**) Hemin attenuated the expression of p53 or p21 stimulated by H_2_O_2_. ^*^P < 0.05 vs. Control; ^#^P < 0.05 vs. H_2_O_2_. n = 5. (**E**) Overexpression of HO-1 infected by recombinant adenovirus decreased the upregulation of p53 or p21 stimulated by H_2_O_2_. ^*^P < 0.05 vs. Ad-GFP; ^#^P < 0.05 vs. Ad-GFP+H_2_O_2_. n = 5. (**F**) Hemin attenuated the expression of γH2A.X (Ser139) stimulated by H_2_O_2_. ^*^P < 0.05 vs. Control; and ^#^P < 0.05 vs. H_2_O_2_. n = 5.

To investigate the effect of HO-1 on endothelial senescence, the expression of HO-1 in HUVECs was induced by the pharmacological inducer Hemin or by the recombinant adenovirus encoding HO-1 with a GFP (Ad-HO-1). As shown in Fig. 1A, the expression of HO-1 was significantly elevated by Hemin and by Ad-HO-1 infection. Endothelial senescence was induced by 50 μM H_2_O_2_ treated for 1 h, followed by incubation with complete culture medium for 2 days. As indicated by Fig. 1B and C, H_2_O_2_ significantly enhanced the proportion of senescence-associated-β-Galactosidase (SA-β-gal)-positive cells, and increased the cell size as observed by the cell morphology, suggesting that endothelial senescence was successfully induced by H_2_O_2_. Treatment of Hemin alone did not alter the senescent level of the HUVECs, but reversed H_2_O_2_-induced endothelial senescence in a dose-dependent manner (Fig. 1B). Additionally, infection of Ad-HO-1 significantly prevented H_2_O_2_-induced senescence of the HUVECs (Fig. 1C).

The cyclin-dependent kinase inhibitors p53 and p21, which lead to inhibition of the cyclin E-CDK2 complex, is regarded as important mechanism underlying endothelial senescence [2]. Indeed, H_2_O_2_ significantly upregulated the expressions of p53 and p21 (Fig. 1D and E). The upregulation of these senescent markers was attenuated by Hemin treatment or HO-1 adenovirus infection (Fig. 1D and E). Thus, the suppression of p53 and p21 by HO-1 is probably the mechanism by which HO-1 ameliorated oxidative stress-induced endothelial aging.

Oxidative stress-induced premature endothelial senescence is also characterized by DNA damage and telomere dysfunction [27]. At the sites of DNA damage and dysfunctional telomeres, the histone variant H2A.X (γH2A.X) is phosphorylated to form DNA damage foci [28,29]. In H_2_O_2_-induced senescent cells, the expression of phosphorylated γH2A.X was augmented (Fig. 1F). Induction of HO-1 by Hemin reverted H_2_O_2_-induced increase of γH2A.X phosphorylation (Fig. 1F), suggesting that HO-1 is able to protect against DNA damage and telomere dysfunction in premature endothelial senescence.

Taken together, these observations indicate that HO-1 ameliorates oxidative stress-induced endothelial senescence.

### HO-1 silencing or HO-1 inhibitor ZnPP exacerbated endothelial senescence

**Figure 2:**
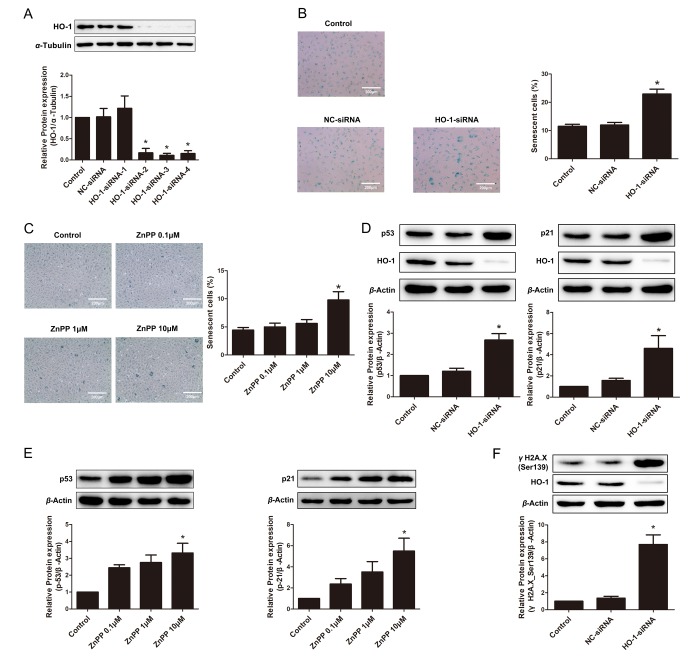
**Endogenous depletion or suppression of HO-1 promoted HUVEC senescence.** (**A**) Screening of HO-1 interference sequences by Western blotting. Sequence-3 was used for the following experiments. ^*^P < 0.05 vs. Control or NC-siRNA. n = 3. (**B**) SA-β-gal staining of HUVEC transfected with HO-1 siRNA (200 × magnification). ^*^P < 0.05 vs. Control or NC-siRNA. n = 5. (**C**) SA-β-gal staining after treating with different concentration of ZnPP (0.1, 1 or 10 μM) (200 × magnification). ^*^P < 0.05 vs. Control. n = 5. (**D**) Silencing of HO-1 increased the expression of p53 or p21. ^*^P < 0.05 vs. Control or NC-siRNA. n = 5. (**E**) HO-1 inhibitor ZnPP increased the expression of p53 or p21. ^*^P < 0.05 vs. Control. n = 5. (**F**) Silencing of HO-1 increased the expression of γH2A.X(Ser139). ^*^P < 0.05 vs. Control or NC-siRNA. n = 5.

The effect of endogenous HO-1 on endothelial senescence was also elucidated by silencing the HO-1 gene. As shown in Fig. 2A, among the four tested siRNAs for HO-1, siRNA-3 demonstrated the best efficiency and thus used for the following studies. Knockdown of endogenous HO-1 markedly increased SA-β-gal-positive HUVECs, implying an exacerbation of endothelial premature aging (Fig. 2B). Similarly, HO inhibitor protoporphyrin IX zinc (II) (ZnPP) dose-dependently induced endothelial senescence, as implied by SA-β-gal staining (Fig. 2C). Moreover, knockdown of endogenous HO-1 enhanced the expressions of senescent markers p53 and p21 (Fig. 2D), as well as the marker of DNA damage- γH2A.X phosphorylation (Fig. 2F). ZnPP, at the concentration of 10 μM, augmented expressions of p53 and p21 (Fig. 2E), indicating that the regulation of endothelial senescence is at least partially dependent on the activity of HO-1.

### HO-1 increased the production of NO in senescent HUVECs

**Figure 3:**
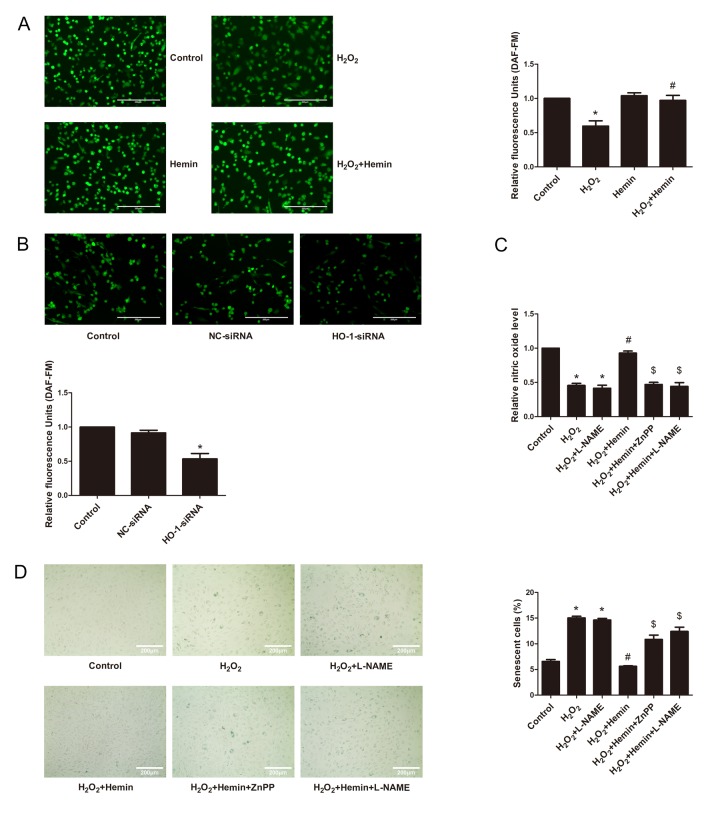
**HO-1 advanced endothelial NO production to resist senescence in HUVECs.** (**A**) HO-1 inducer Hemin reversed the reduction of the relative fluorescence intensity of DAF-FM indicated endothelial NO production stimulated by H_2_O_2_ (200 × magnification). The fluorescence intensity of DAF-FM was normalized to the cell numbers by normalizing to DAPI fluorescence (not shown). ^*^P < 0.05 vs. Control; and ^#^P < 0.05 vs. H_2_O_2_. n = 5. (**B**) Silencing of HO-1 decreased the relative fluorescence intensity of DAF-FM indicated endothelial NO production (200 × magnification). The fluorescence intensity of DAF-FM was normalized to the cell numbers by normalizing to DAPI fluorescence (not shown). ^*^P < 0.05 vs. Control or NC-siRNA. n = 5. (**C**) NO production was measured according to Griess assay. Hemin reversed the reduction of endothelial NO production stimulated by H_2_O_2_, while the protective effect was abolished by ZnPP or L-NAME. ^*^P < 0.05 vs. Control; ^#^P < 0.05 vs. H_2_O_2_; ^$^P < 0.05 vs. H_2_O_2_+Hemin. n = 5. (**D**) SA-β-galactosidase staining results showed that ZnPP or L-NAME abolished the anti-senescence effect of Hemin (200 × magnification). ^*^P < 0.05 vs. Control; ^#^P < 0.05 vs. H_2_O_2_; ^$^P < 0.05 vs. H_2_O_2_+Hemin. n = 5.

Impairment of NO production is a hallmark of endothelial aging [30–34]. In the present study, DAF-FM probe was used to detect endothelial NO production [35]. As shown in Fig. 3A, the fluorescence intensity of NO was reduced by H_2_O_2_ treatment, but was reversed by Hemin treatment. Contrarily, silencing of HO-1 decreased fluorescence intensity of DAF-FM (Fig. 3B). NO production was also measured according to Griess assay. As shown in Fig. 3C, Hemin reversed the reduction of endothelial NO production stimulated by H_2_O_2_, while this protective effect was abrogated by HO-1 inhibitor ZnPP or eNOS inhibitor N^ω^-nitro-l-arginine methyl ester (L-NAME). Moreover, SA-β-galactosidase staining results showed that ZnPP or L-NAME abolished the anti-senescent effect of Hemin (Fig. 3D). It’s thus suggested that HO-1 is able to preserve the reduction of NO production in endothelial senescence, which depends on eNOS activation.

### HO-1 potentiated eNOS phosphorylation at serine 1177 via Akt

**Figure 4:**
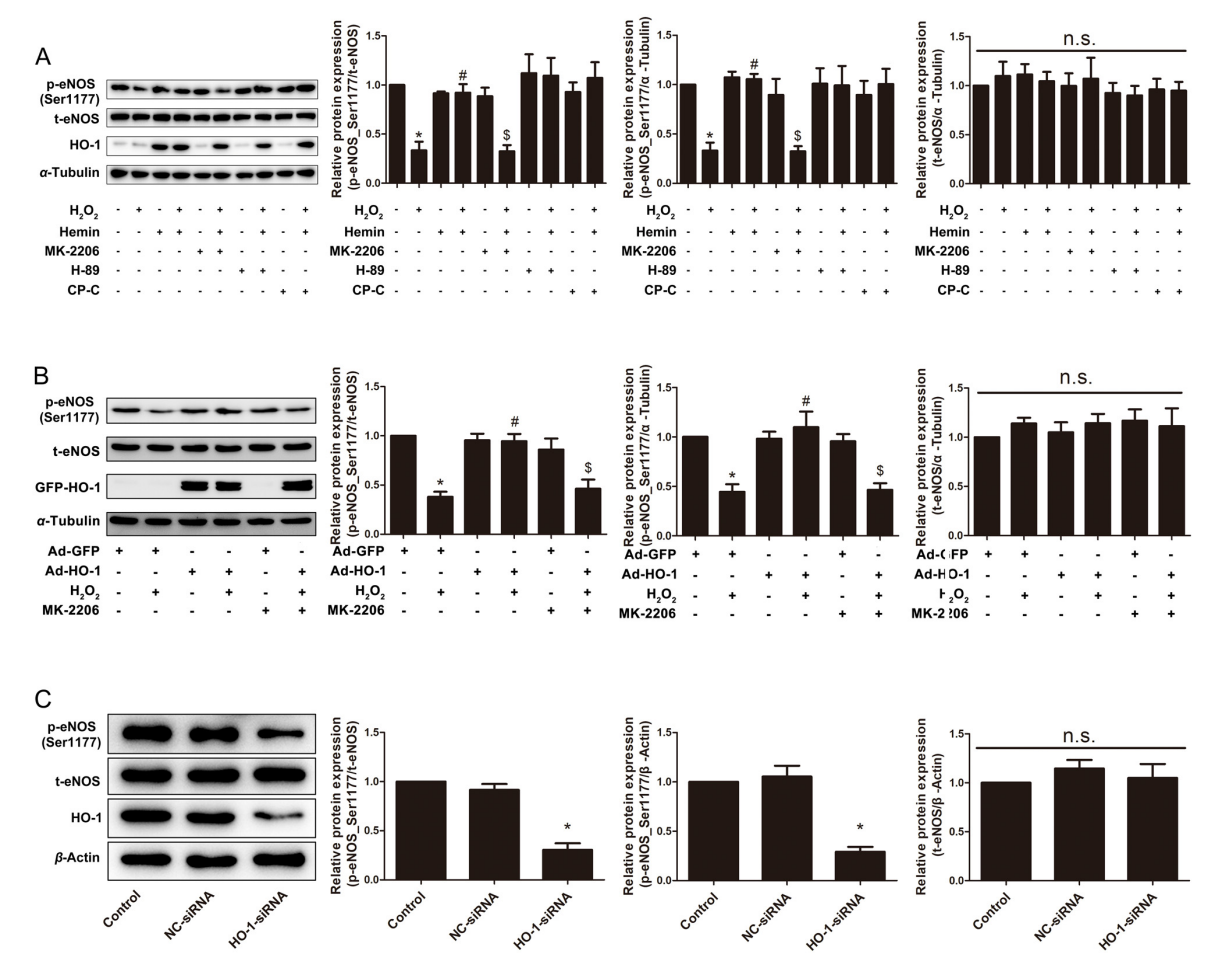
**HO-1 retarded H_2_O_2_-induced HUVEC senescence through eNOS activation via Akt.** (**A**) Expressions of p-eNOS(Ser1177) and total eNOS were measured in HUVECs treated with or without H_2_O_2_ and Hemin in the presence or absence of Akt inhibitor MK-2206, PKA inhibitor H-89 or AMPK inhibitor Compound C (CP-C). ^*^P < 0.05 vs. Control; ^#^P < 0.05 vs. H_2_O_2_; ^$^P < 0.05 vs. H_2_O_2_+Hemin; n.s., non significant. n = 5. (**B**) Expressions of p-eNOS(Ser1177) and total eNOS were measured in HUVECs infected with or without HO-1 recombinant adenovirus in the presence or absence of Akt inhibitor MK-2206. ^*^P < 0.05 vs. Ad-GFP; ^#^P < 0.05 vs. Ad-GFP+H_2_O_2_; ^$^P < 0.05 vs. Ad-HO-1+H_2_O_2_; n.s., non significant. n = 5. (**C**) Expressions of p-eNOS(Ser1177) and total eNOS were measured in HUVECs transfected with or without HO-1 siRNA. ^*^P < 0.05 vs. Control or NC-siRNA; n.s., non significant. n = 5.

The activation of eNOS, resulting in sustaining generation of NO, is crucial for maintaining the endothelial function. The activation of eNOS is characterized by phosphorylation at serine 1177 and dephosphorylation at threonine 495 [36–38]. In senescent HUVECs induced by H_2_O_2_, the phosphorylation level of eNOS at Ser1177 was significantly decreased, but the total expression level of the protein was not altered (Fig. 4A and B). Both the HO-1 inducer Hemin and infection with Ad-HO-1 rendered the rebound of the phosphorylation of Ser1177 (Fig. 4A and B). By contrast, silencing of endogenous HO-1 inhibited eNOS phosphorylation at Ser1177, without affecting expression of total eNOS (Fig. 4C). These results indicate that HO-1 prevents eNOS inactivation in aging endothelial cells, and that HO-1 insufficiency triggers premature senescence. Phosphorylation of Thr495, the negative regulatory site of eNOS, was unchanged in H_2_O_2_-induced senescent endothelial cells, and was also unaltered by Hemin or HO-1 siRNA (Supplementary Fig. 1).

The phosphorylation status of a protein depends on the balanced actions of protein kinases and phosphatases. Many kinases have been identified to phosphorylate eNOS at a number of consensus motifs, including protein kinases A (PKA) [39], B (Akt) [40], and C [41], AMP-activated kinase (AMPK) [42,43], calmodulin kinase II [44]. Among all, the Phosphatidylinositol 3-kinase (PI3K)-Akt signaling pathway is the predominant upstream kinases responsible to eNOS phosphorylation at serine 1177 residue [45,46]. As shown in Fig. 4A, the restored phosphorylation of Ser1177 by Hemin was abolished by Akt inhibitor MK-2206, rather than PKA inhibitor H-89 or AMPK inhibitor Compound C. Besides, MK-2206 reversed the restored phosphorylation of Ser1177 by Ad-HO-1 (Fig. 4B), supporting the conclusion that HO-1 enhances eNOS activation via an Akt-dependent manner. However, treatment with Hemin did not change either the phosphorylation status of Akt at threonine 473 and threonine 308, or the total expression of Akt (Supplementary Fig. 2), implying that HO-1 does not directly alter Akt activation or expression.

Reportedly, protein phosphatase 2A (PP2A) has been known to dephosphorylate eNOS at serine 1177 and ultimately inhibit the activity of eNOS [47,48]. Nevertheless, in our experiments, neither the expression nor the activity of PP2A was altered by HO-1 inducer Hemin or HO-1 silencing (Supplementary Fig. 3). These observations thus suggest that the augmented eNOS phosphorylation at serine 1177 by HO-1 is not attributed to the alterations of PP2A.

### HO-1 enhanced interactions between eNOS and Akt

**Figure 5:**
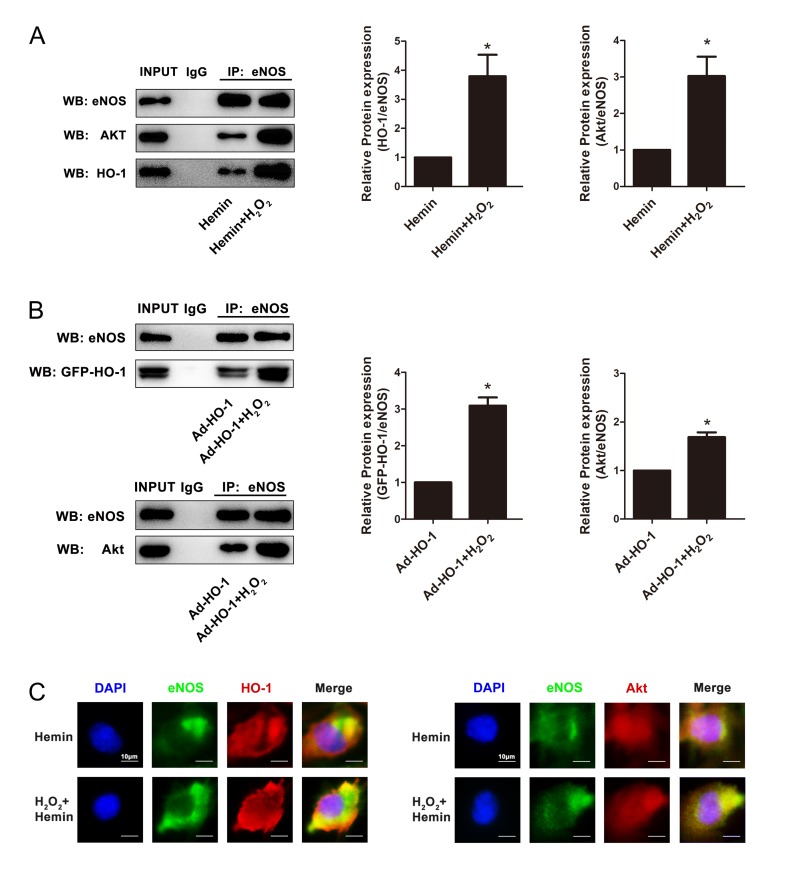
**HO-1 regulated endothelial senescence induced by H_2_O_2_ through HO-1-eNOS-Akt interaction.** (**A**) Overexpression of HO-1 induced by Hemin enhanced the interaction between HO-1 and eNOS, and promoted the interaction between eNOS and Akt in senescent HUVECs induced by H_2_O_2_. ^*^P < 0.05, Hemin+H_2_O_2_ vs. Hemin. n = 5. (**B**) Overexpression of HO-1 mediated by HO-1 recombinant adenovirus enhanced the interaction between HO-1 and eNOS, and promoted the interaction between eNOS and Akt in senescent HUVECs induced by H_2_O_2_. ^*^P < 0.05, Ad-HO-1+H_2_O_2_ vs. Ad-HO-1. n = 5. (**C**) Overexpression of HO-1 enhanced the colocalization between HO-1 and eNOS, and promoted the colocalization between eNOS and Akt (400 × magnification) in senescent HUVECs induced by H_2_O_2_.

Since HO-1 did not affect the expression or activity of the key kinase Akt, it is hypothesized that the interaction of Akt and eNOS is enhanced by HO-1. Co-IP experiments suggested that HO-1 and eNOS had physical interactions. Overexpression of HO-1 promoted the interaction between HO-1 and eNOS, as well as the interaction between eNOS and Akt in senescent HUVECs induced by H_2_O_2_ (Fig. 5A and B). Similarly, immunofluorescence assay confirmed that Hemin enhanced the co-localization between HO-1 and eNOS, as well as the co-localization between eNOS and Akt in senescent HUVECs induced by H_2_O_2_ (Fig. 5C). These observations raised the possibility that HO-1 might promote the activity of Akt enhancing interaction between Akt with eNOS.

### HO-1 prevented eNOS uncoupling

**Figure 6:**
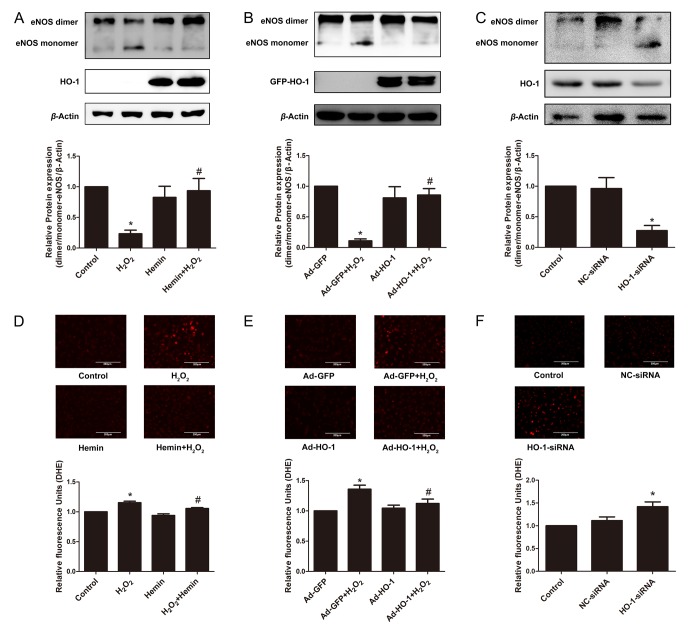
**HO-1 protected the coupling state of eNOS in endothelial senescence.** (**A**) HO-1 inducer Hemin increased the downregulation of dimer/monomer-eNOS ratio stimulated by H_2_O_2_. ^*^P < 0.05 vs. Control; and ^#^P < 0.05 vs. H_2_O_2_. n = 6. (**B**) Overexpression of HO-1 infected by recombinant adenovirus increased the downregulation of dimer/monomer-eNOS ratio stimulated by H_2_O_2_. ^*^P < 0.05 vs. Ad-GFP; and ^#^P < 0.05 vs. Ad-GFP+H_2_O_2_. n = 3. (**C**) Silencing of HO-1 decreased the dimer/monomer-eNOS ratio. ^*^P < 0.05 vs. Control or NC-siRNA. n = 5. (**D**, **E** and **F**) Images of DHE fluorescence staining taken by confocal microscopy showing ROS production in HUVECs (200 × magnification). The fluorescence intensity of DHE was normalized to the cell numbers by normalizing to DAPI fluorescence (not shown). (**D**) treated with or without H_2_O_2_ and Hemin. ^*^P < 0.05 vs. Control; and ^#^P < 0.05 vs. H_2_O_2_. n = 4. (**E**) infected with or without HO-1 recombinant adenovirus. ^*^P < 0.05 vs. Ad-GFP; and ^#^P < 0.05 vs. Ad-GFP+H_2_O_2_. n = 6. (**F**) transfected with or without HO-1 siRNA. ^*^P < 0.05 vs. Control or NC-siRNA. n = 6.

The formation of eNOS dimer is required for the normal function of eNOS, whereas uncoupled eNOS produces peroxynitrite (ONOO^−^) rather than NO [38,49]. To investigate the effect of HO-1 on eNOS coupling, the expression of eNOS dimer and monomer were compared. As shown in Fig. 6A and B, the ratio of eNOS dimer to monomer was reduced in aging cells induced by oxidative stress, but was overturned after the treatment of Hemin or HO-1 adenovirus. In contrary, knockdown of endogenous HO-1 potentiated eNOS uncoupling, as implied by the decreased ratio of eNOS dimer to monomer (Fig. 6C). Furthermore, Hemin or HO-1 adenovirus reduced the content of intracellular ROS stimulated by H_2_O_2_, while silencing of HO-1 increased ROS generation (Fig. 6D, E and F). Taken together, these observations indicate that HO-1 may prevent eNOS uncoupling by inhibiting ROS generation in endothelial senescence.

### HO-1 alleviated *in vivo* endothelial senescence in the aorta of SHRs

**Figure 7:**
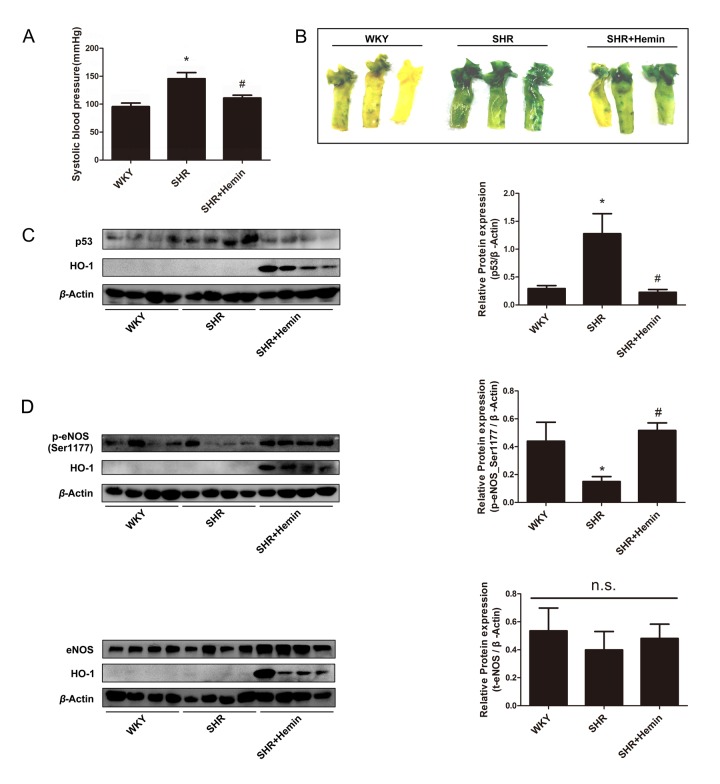
**HO-1 alleviated endothelial senescence through regulating phosphorylation status of eNOS at Ser1177 in SHRs.** (**A**) Upregulation of HO-1 reduced blood pressure in the SHR. ^*^P < 0.05 vs. WKY; ^#^P < 0.05 vs. SHR. n = 6. (**B**) SA-β-gal staining of the aortas originated from WKYs, SHRs, and SHRs treated with Hemin. (**C**) Hemin attenuated the high expression of p53 in the SHR. ^*^P < 0.05 vs. WKY; ^#^P < 0.05 vs. SHR. n = 8. (**D**) Hemin increased the downregulation of p-eNOS (Ser1177) in SHRs, while had no effect on the expression of eNOS. ^*^P < 0.05 vs. WKY; ^#^P < 0.05 vs. SHR; n.s., non significant. n = 7-12.

In SHRs, endothelial premature senescence and dysfunction are caused by a consistently elevated arterial blood pressure [50]. Thus, SHR is considered to a typical *in vivo* model of endothelial senescence. 12-week-old SHRs received a 10-day treatment of Hemin to observe the *in vivo* effect of HO-1. The age-matched WKYs were served as the normotensive control group, while the untreated SHRs were served as the model group. At the end of treatment, all the animals were sacrificed, and the aortas were isolated for SA-β-gal staining and Western blot analysis. Upregulation of HO-1 induced by Hemin reduced blood pressure in the SHRs (Fig. 7A). As shown in Fig. 7B, the aortas of SHRs had significantly higher SA-β-gal blue-staining level compared to WKY controls. In aortas of Hemin-treated SHRs, a significant decrease in SA-β-gal staining signal was observed as compared to that of the untreated-SHRs. Additionally, Hemin treatment decreased the expression of p53 in SHRs (Fig. 7C). As shown in Fig. 7D, Hemin reversed the downregulation of p-eNOS (Ser1177) in SHRs, while didn’t affect the expression of total eNOS. Consistently with the *in vitro* experiments, these results confirm that HO-1 prevents endothelial senescence through regulating eNOS.

## DISCUSSION

Premature senescence of vascular endothelial cells induced by oxidative stress is a hallmark of the decline of endothelial function, and has been proposed to be involved in the development of various cardiovascular diseases such as hypertension and atherosclerosis [1,2,51–53]. Therapeutic strategies targeting endothelial senescence have shed new lights on the treatment of cardiovascular diseases [7]. The present study revealed that HO-1 ameliorates H_2_O_2_-induced premature endothelial senescence and is considered to be a promising target. This conclusion is firstly supported by the observations that treatment with HO-1 inducer Hemin or infection of HO-1 adenovirus prevented H_2_O_2_-induced expression of cell senescence marker SA-β-gal, upregulation of cell cycle inhibitor p53 and p21, as well as increase of phosphorylated γH2A.X level which indicates DNA damage and telomere dysfunction. Additionally, the observations that depletion of endogenous HO-1 by siRNA or treatment with ZnPP, the competitive inhibitor of HO-1 which inhibits HO activity with or without altering its expression [54], enhanced expressions of these senescent markers, further confirm that deficiency of endogenous HO-1 promoted endothelial senescence and that the effect of HO-1 is at least partially dependent on its activity. In line with the findings that activation of the Nrf2-dependent cellular anti-oxidant defense system prevents endothelial senescence (Supplementary Fig. 4) [55–57], and that inhibition of Nrf2 significantly promotes cellular senescence [58,59], these findings suggest that HO-1, as one of the most important downstream target genes of Nrf2, contributes to the anti-senescent effects of Nrf2.

Mechanistically, the effect of HO-1 on endothelial senescence is most likely due to its regulation in NO production. Indeed, the impairment of NO release and decrease of NO bioavailability have been implicated in senescent endothelial cells [31–34,51]. Treatment with NO donor DETA-NO or transfection with eNOS protects against the development of endothelial senescence [60–62], whereas the eNOS inhibitor L-NAME or Asymmetrical dimethylarginine (ADMA) ab [56] abrogates the anti-senescent effect of NO and elicits endothelial and vascular senescence [60,61,63,64]. In this study, NO production detected by DAF-FM fluorescent probe and Griess assay was significantly enhanced by Hemin treatment, but demonstrated a reduction by HO-1 knockdown, suggesting that HO-1 is able to influence the generation of NO. In addition, L-NAME abolished the protective effect of Hemin on augmenting NO production and preventing endothelial senescence, further supporting the conclusions that the anti-senescent effect of HO-1 is attributed to its improvement in NO generation. The mechanisms underlying the regulation of NO in endothelial aging are still undetermined. Some studies indicate that NO enhances telomerase activity, as implied by the results that eNOS transfection and NO donor are comparable to hTERT transfection in increasing telomerase activity and inhibiting cellular senescence [60,62]; whereas a study provides evidence challenging that physiological concentrations of NO could modulate telomerase levels [65]. Additionally, it has been suggested that defective eNOS activity, which leads to a gradual reduction of NO bioavailability but an increased formation of harmful free radicals including peroxynitrite (ONOO^−^) and N_2_O_3_ in the vasculature, causes DNA and telomere damage to facilitate endothelial aging [66,67]. The damage of genomic stability is characterized by the phosphorylation of the γH2A.X to form DNA damage foci, the stabilization of p53 through phosphorylation, the increase in the expression of its downstream transcriptional target p21, and finally inhibition of the cyclin E-CDK2 complex [2]. Thus, the observations of the present study that upregulation or overexpression of HO-1 diminished oxidative stress-induced phosphorylation of γH2A.X and expressions of p53 and p21, meanwhile HO-1 deficiency or inhibition trigger these responses of DNA and telomere damage, might suggest that eNOS activation by HO-1 is probably involved in its anti-senescent properties.

Since the activation of eNOS results from the phosphorylation of the enzyme [37,38,68], the phosphorylation level of eNOS was firstly determined to find out the answer how HO-1 modulates eNOS activation and NO production. The most important phosphorylation sites of eNOS are serine 1177 and threonine 495. The former can increase the Ca^2+^ sensitivity and represent an additional and independent mechanism of eNOS activation; while the latter is a negative regulatory site, the phosphorylation of which can interfere with the binding of calmodulin [37,38]. In the presence of the essential cofactor tetrahydrobiopterin (BH4), the activated eNOS oxidizes its substrate L-arginine to form NO and L-citrulline [69]. A major finding of our study is that HO-1 potentiated eNOS phosphorylation at Ser1177 to enhance eNOS activation. This conclusion is supported by the results that Hemin treatment or Ad-HO-1 infection reversed the decreased eNOS phosphorylation at Ser1177 induced by H_2_O_2_ while HO-1 silencing restrained this phosphorylation, and that HO-1 did not alter either the total expression of eNOS nor the phosphorylation at Thr495. Nevertheless, HO-1 is not a kinase or phosphatase, so it seems impossible that HO-1 activates eNOS directly. Among all the kinases and phosphatases responsible to eNOS phosphorylation at Ser1177, Akt is the predominant kinase [45] and PP2A is the most important phosphatase [47,48]. Thus, we detected the activation of Akt and PP2A in HUVECs with HO-1 induction or knockdown. Our work showed that HO-1 did not affect either the phosphorylation of Akt at Ser473 or Thr308, the total expression of Akt, nor the activity or expression of PP2A. Therefore, it would rule out the possibility that HO-1 influences eNOS activation through altering its upstream kinases or phosphatases. Surprisingly, we discovered that there was a protein-protein interaction between HO-1 and eNOS, as implied by the co-IP and immunofluorescence results. Overexpression of HO-1 enhanced the co-localization and interaction of HO-1 and eNOS, and advanced the interaction between eNOS and Akt. We speculate that the binding of HO-1 to eNOS might alter the space conformation of eNOS, enhancing its interaction with Akt, subsequently facilitating the phosphorylation of eNOS by Akt. In fact, similar interactions between heat shock protein 90 (Hsp90) and eNOS or nNOS have been identified, which could activate the NOSs by modulating their phosphorylation or binding with Ca^2+^/calmodulin [70–72]. Since HO-1 is also regarded as a heat shock protein 32 (Hsp32), for its promoter regions containing heat shock elements similar to those identified in the regulatory regions of various heat shock protein genes [73], it is possible that HO-1 shares conformational and functional similarity with Hsp90 in the regulation of the NOS activity.

In addition, uncoupling of eNOS is an important mechanism of oxidative stress and decreased NO bioavailability in endothelial and vascular senescence [27,74,75]. In the aging endothelial cells, increased amount of ·O_2_^−^ reacts with NO to form ONOO^−^, which leads to eNOS uncoupling via oxidation of BH4 [74,76]. The uncoupled eNOS shifts the nitroso-redox balance favoring production of ·O_2_^−^ rather than NO, resulting in further increased formation of endothelial ROS and the activation of redox-sensitive genes that contribute to endothelial dysfunction [74,76–79]. Indeed, our experiments demonstrated eNOS uncoupling in senescent HUVEC model induced by H_2_O_2_. Interestingly, HO-1 induction or overexpression reduced the formation of uncoupled eNOS, whereas silencing of endogenous HO-1 triggered eNOS uncoupling. Since HO-1 is a potent antioxidant enzyme against ROS, as demonstrated by the DHE fluorescence experiments, it is most likely that HO-1 diminished ROS production to prevent eNOS uncoupling in endothelial senescence.

The effect of HO-1 on endothelial senescence was also investigated *in vivo* by using SHRs as a model. In SHRs, chronic high blood pressure induces endothelial dysfunction, as demonstrated by decrease of NO bioavailability, impairment of endothelium-dependent hyperpolarization, and facilitation of endothelium-dependent contractions [1,80,81]. NO donor exerted anti-hypertensive effects in SHRs through the restoration of vascular endothelial protective functions [82]. Our findings demonstrated that endothelium of aortas collected from 12-week-old SHRs presented a severe senescent phenotype, as implied by SA-β-gal staining results and p53 expression level, and as compared with the age-matched normotensive control WKYs. Treatment of HO-1 pharmacological inducer Hemin for 10 days ameliorated endothelial senescence in SHRs aortas. In addition, induction of HO-1 alleviated endothelial senescence through regulating phosphorylation status of eNOS at Ser1177. The present findings are in line with the *in vitro* results and confirm that induction/overexpression of HO-1 prevents endothelial aging induced by hypertension. Notably, upregulation of HO-1 lowers arterial blood pressure in the SHRs [17–21]. The inhibitory effect of HO-1 against endothelial senescence via regulating eNOS might contribute to its improvement of endothelial function and anti-hypertensive properties. Interestingly, the mechanisms by which HO-1 protects against endothelial dysfunction seem to differ from different stages of hypertension. In 12 weeks old SHRs which are used in the present study, HO-1 mainly enhances eNOS activation and inhibits eNOS uncoupling to reverse endothelial premature aging; whereas in 36 weeks old SHRs, up-regulation of HO-1 reverses endothelial dysfunction via impairing endothelium-dependent contractions and enhancing endothelium-dependent hyperpolarization, without affecting NO-mediated vasodilatations [20,21]. This discrepancy might probably due to the dysfunction of eNOS and impaired responsiveness of sGC to NO in the old SHRs [83–85].

Intriguingly, as an inducible enzyme, the expression of HO-1 was induced during the development of endothelial aging resulted from H_2_O_2_ treatment (Supplementary Fig. 5A). Taken into account that HO-1 exerts protective effects against endothelial senescence, this induction of HO-1 might be an intracellular compensatory mechanism against oxidative stress-induced endothelial aging. However, it is important to note that compensatory induction of HO-1 was not sustained and was attenuated 24 hours after H_2_O_2_ stimulation (Supplementary Fig. 5A). Moreover, the expression of HO-1 was observed to be decreased in the senescent HUVECs (Passage 11 of sub-cultured cells) as compared with the young cells (Passage 3) (Supplementary Fig. 5B). These observations thus suggest that the induction of HO-1 as an endogenous cellular compensation against senescence is limited. Pharmacological inducers of HO-1 or gene therapy delivery of HO-1 gene, which lead to sustained induction or upregulation of HO-1, might be more promising for therapeutic use. Since most of the currently available pharmacological inducers of HO-1, in particular the heavy metals, show cellular and tissue toxicity [86], there is a need to develop new HO-1 inducers that are effective and safe. Natural compounds which show a potent effect on HO-1 induction or enhancement of the upstream Nrf-2/HO-1 pathway may be promising sources. Moreover, the application of genetic interventions may allow selective tissue targeting of HO-1 induction, and may lead to the development of more effective and long-lasting therapies for cardiovascular diseases such as hypertension and its vascular complications.

**Figure 8:**
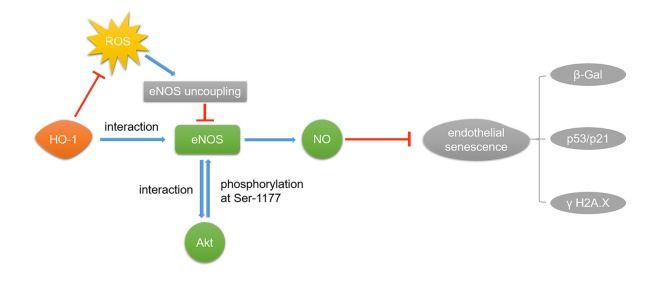
**HO-1 ameliorates H_2_O_2_-induced premature senescence in HUVECs via eNOS.** HO-1 attenuates H_2_O_2_-induced expressions of p53/p21 and ameliorated oxidative stress-induced DNA damage in endothelia senescence through two mechanisms: 1) HO-1 prevents eNOS uncoupling by inhibiting ROS. 2) Interaction between HO-1 and eNOS makes eNOS easier to interact with Akt, for which Akt can phosphorylate eNOS at Ser1177.

In summary, the present study suggests that HO-1 ameliorates oxidative stress-induced premature senescence in HUVECs via regulating eNOS through the following mechanisms: 1) HO-1 interacts with eNOS to enhance the interaction between eNOS and Akt, and then improves eNOS phosphorylation at Ser1177; 2) HO-1 provides antioxidant protection to endothelial cells and maintains the stable state of eNOS dimer, finally resulting in increased production of endothelial NO (Fig. 8). These findings provide important new insights into the effect of HO-1 on treating endothelial senescence and dysfunction in hypertension or other cardiovascular diseases.

## MATERIALS AND METHODS

### Cell culture

Neonatal umbilical cords were collected from The First Affiliated Hospital, Sun Yat-sen University. Human umbilical vein endothelial cells (HUVECs) were isolated from fresh human umbilical veins using 0.25% Trypsin. HUVECs were grown in endothelial cell medium (ECM, ScienCell, USA) supplemented with 5% fetal bovine serum (FBS), 1% penicillin/streptomycin and 1% endothelial cell growth supplement (ECGS). The cells were maintained at 37 °C in humidified 5% CO_2_. Senescent HUVECs (passage 3-8) were induced by H_2_O_2_ (50 μM, incubated for 1 h, followed by cultured with fresh ECM for 48 h), as judged by senescence-associated β-galactosidase (SA-β-gal) assay and cell morphological appearance. Hemin (Sigma-Aldrich, St. Louis, MO) was used to evaluate the effect of HO-1 on endothelial senescence, and Akt inhibitor MK-2206 (Selleck, Texas, USA), PKA inhibitor H-89 (MedChemExpress, New Jersey, USA) and AMPK inhibitor Compound C (Selleck, Texas, USA) were used to investigate the potential involvement of Akt, PKA and AMPK in the effect of HO-1.

### Animals

Animal procedures used in this study were in accord with institutional guidelines and were approved by Laboratory Animal Center of the Sun Yat-sen University. 12-week-old Wistar-Kyoto rats (WKYs) and spontaneously hypertensive rats (SHRs) were purchased from Beijing Vital River Laboratory Animal Technology CO. The animals were randomly divided into 3 groups. The treated group (SHRs, n = 12) received a single intraperitoneal injection of Hemin (10 mg·kg^-1^ per day, Sigma-Aldrich, St. Louis, MO) for 10 days. The control group (WKYs, n = 12) and the model group (SHRs, n = 12) were injected with normal saline. After 10 days, all the rats were euthanized and aortas were collected and subjected to staining or Western blot analysis.

### Senescence-associated-β-Galactosidase Staining (SA-β-gal staining)

*In situ* staining for SA-β-galactosidase was performed by using a senescence detection kit (Beyotime Biotechnology, Shanghai, China). Briefly, the cells were incubated in freshly prepared SA-β-gal staining solutions at 37 °C for 24 h. Blue staining indicating the presence of SA-β-gal was detected under the microscope. To calculate the percentage of SA-β-gal-positive cells, stained cells were counted and related to the total cell number. Similarly, the rat aortas obtained from the euthanized WKYs and SHRs were subjected to β-gal staining as described as above-mentioned.

### Adenovirus infection

HUVECs were infected with recombinant adenovirus encoding human HO-1 (Vigenebio, Shandong, China) to overexpress HO-1. Recombinant adenovirus of GFP was used as the negative vector.

### RNA interference

HUVECs were transfected at 80% confluence with 20 nM HO-1-siRNA (CCAGCAACAAAGTGCAAGA, Ribobio, Guangzhou, China) or with Non-targeting Control Pool siRNA (NC-siRNA) in Opti-MEM (Gibco, Grand Island, NY), using Lipofectamine 2000 (Invitrogen). The medium was changed to ECM after transfection for 5 h, and the cells were maintained for 48 h before further experiments.

### Western blot analysis and low temperature SDS-PAGE

Equal amounts (30 μg) of protein were subjected to SDS gel electrophoresis (8-12% SDS/polyacrylamide gel) for approximately 80 min at 110 V and transferred to PVDF membrane by wet electro-blotting (230 mA, 2 h) using the standard Western blot protocol. Immune-reactive proteins were detected using an enhanced chemiluminescence system (ECL+, Tanon, Shanghai, China).

Low-temperature SDS-PAGE was performed for detection of eNOS monomers and dimers. In brief, total proteins were incubated in loading buffer without 2-mercaptoethanol at 37 °C for 5 min and subsequently subjected to 8% SDS-PAGE for 4 h at a constant current of 40 mA, and the buffer tank was placed in an ice bath during electrophoresis.

The following antibodies were used: mouse monoclonal anti-eNOS (Pharmingen BD Biosciences, USA), mouse monoclonal anti-phospho-eNOS (Ser1177, Pharmingen BD Biosciences, USA), rabbit polyclonal anti-phospho-eNOS (Thr495, Cell Signaling Technology Danvers, MA), mouse monoclonal anti-phospho-Akt (Ser473, Cell Signaling Technology Danvers, MA), rabbit polyclonal anti-Akt (Cell Signaling Technology Danvers, MA), rabbit polyclonal anti-p53 (Proteintech, Manchester, UK), rabbit polyclonal anti-p21 (Proteintech, Manchester, UK), rabbit polyclonal anti-HO-1 (Proteintech, Manchester, UK), rabbit polyclonal anti-H2A.X (Ser139, Abcam, Cambridge, UK), rabbit polyclonal anti- phospho-Akt (Thr308, Abcam, Cambridge, UK), mouse monoclonal anti-β-Actin (Proteintech, Manchester, UK), mouse monoclonal anti-α-Tubulin (Sigma-Aldrich, St. Louis, MO). And secondary antibodies polyclonal antibodies purchased from Cell Signaling were used.

### Co-immunoprecipitation (co-IP)

300 μg lysate was used for immunoprecipitation, whereas 20 μg was used for input or direct immunoblotting. For anti-eNOS-immunoprecipitations, rabbit polyclonal anti-eNOS (Santa Cruz, California, USA) was added to the cell lysates and incubated with rotation overnight at 4 °C. And 25 μl of protein A/G beads (Thermo Fisher, New York, USA) were added to the mixed solution. After incubation for 4 h at 4 °C, the beads were washed 3 times with lysis buffer containing 150-500 mM NaCl. Beads were incubated in the final salt wash for 5 min to reduce non-specific binding. Immunoprecipitated proteins were denatured by the addition of 25 μl of 2 × loading buffer and boiled for 5 min, resolved by 10% SDS-PAGE, and analyzed by immunoblotting.

### Immunofluorescence

HUVECs were cultured on 48-well chamber slides and stimulated with H_2_O_2_ (50 μM) for 1 h and grown for 48 h to induce senescent cells. Cells were then treated with 10 μM Hemin or the vehicle (Saline) for 48 h. After 2 days, cells were fixed with 4% (w/v) paraformaldehyde for 30 min, washed with PBS for 5 min, and permeabilized in 0.1% (w/v) Triton X-100 at room temperature for 10 min. The cells were incubated with mouse monoclonal antibody against eNOS (1:50), rabbit polyclonal antibody against HO-1 (1:100) or rabbit polyclonal antibody against Akt (1:50) at 4 °C overnight. After incubation with Alexa Fluor 594-conjugated anti-rabbit IgG (H+L) secondary antibody and Alexa Fluor 488-conjugated anti-mouse IgG (H+L) secondary antibody (Proteintech, Manchester, UK) for 1 h at room temperature, nuclei were stained with DAPI (5 mg/ml, Sigma-Aldrich, St. Louis, MO) and analyzed by cell auto imaging system (EVOS FL Auto, Life Technologies, New York, USA).

### Phosphatase activity assay

Protein phosphatase 2A (PP2A) activity was determined using the PP2A immunoprecipitation phosphatase assay kit (Millipore, Temecula, USA). HUVECs were scraped off the dishes with 0.3 ml of phosphatase extraction buffer containing 20 mM imidazole-HCl, 2 mM EDTA, 2 mM EGTA, pH 7.0, with a protease inhibitor tablet and 1 mM PMSF. The cells were sonicated for 10 s and centrifuged at 2000 g for 5 min, and the supernatants were used for the assay of phosphatase activities. Cell extracts containing equivalent amounts of protein (50-200 μg) brought volume to 500 μl with pNPP Ser/Thr Assay Buffer were then incubated 2 h at 4 °C in the presence of 4 μg anti-PP2A C subunit antibody and 30 μl Protein A agarose slurry. The pellets were washed three times with 700 μl TBS, followed by one wash with 500 μl pNPP Ser/Thr Assay Buffer. The pellets added with 60 μl diluted phosphopeptide(K-R-pT-I-R-R) and 20 μl pNPP Ser/Thr Assay Buffer were then incubated for 10 min at 30 °C in a shaking incubator. Centrifuge briefly and transfer 25 μl into reaction solution. Activities of PP2A were determined by using a malachite green phosphatase assay protocol followed by the measurement of absorbance at 650 nm.

### Measurement of ROS

Living HUVECs were incubated for dye uptake with were 10 μM 2,7-Diamino-10-ethyl-9-phenyl-9,10-dihydrophenanthridine (DHE, Sigma-Aldrich, St. Louis, MO) with cell auto imaging system. The fluorescence intensity was determined by High-Content Screening instrument (ArrayScan VTI 600 plus, Thermo Fisher, New York, USA), and was normalized to the cell numbers by normalizing to DAPI fluorescence (for nucleus staining).

### Measurement of endothelial NO production

The production of NO was measured using the NO-specific fluorescent dye 3-Amino-4-aminomethyl-2',7'-difluorescein, diacetate (DAF-FM DA, Beyotime Biotechnology, Shanghai, China). In brief, the cells were loaded with 5 μM DAF-FM DA for 30 min at 37 °C and rinsed three times with M199 (Gibco, Grand Island, NY). Then the cells were visualized and photographed using cell auto imaging system. The images were analyzed by Image J Software Version 1.40 (National Institutes of Health, USA). The fluorescence intensity of DAF-FM was normalized to the cell numbers by normalizing to DAPI fluorescence (for nucleus staining).

### Measurement of NO production in culture medium

The HUVECs were seeded and cultured in 24-well plates. After treatment, the medium (500 μl) of each well was collected in microcentrifuge tubes. NO content present in the medium was evaluated by measuring nitrite according to the Griess assay using Total Nitric Oxide Assay Kit (Beyotime Biotechnology, S0023). The optical density was measured at 540 nm. The amount of nitrite in the culture media was calculated using sodium nitrite as a reference standard.

### Statistical analysis

Data were presented as means ± SEM, and analyzed by two-tailed unpaired Student’s t-test between two groups and by one-way ANOVA followed by the *Bonferroni post hoc* test for multiple comparisons using GraphPad Prism Software Version 5.01 (La Jolla, CA). P < 0.05 was considered to be statistically significant.

## Supplementary Material

Supplementary Figure 1

Supplementary Figure 2

Supplementary Figure 3

Supplementary Figure 4

Supplementary Figure 5
