# Supplementary figures and images for: Heme oxygenase-1 ameliorates oxidative stress-induced endothelial senescence via regulating endothelial nitric oxide synthase activation and coupling
Source: Aging (Albany NY). 2018 Jul 24;10(7):1722–44. doi: 10.18632/aging.101506 (PMC6075439; doi:10.18632/aging.101506)

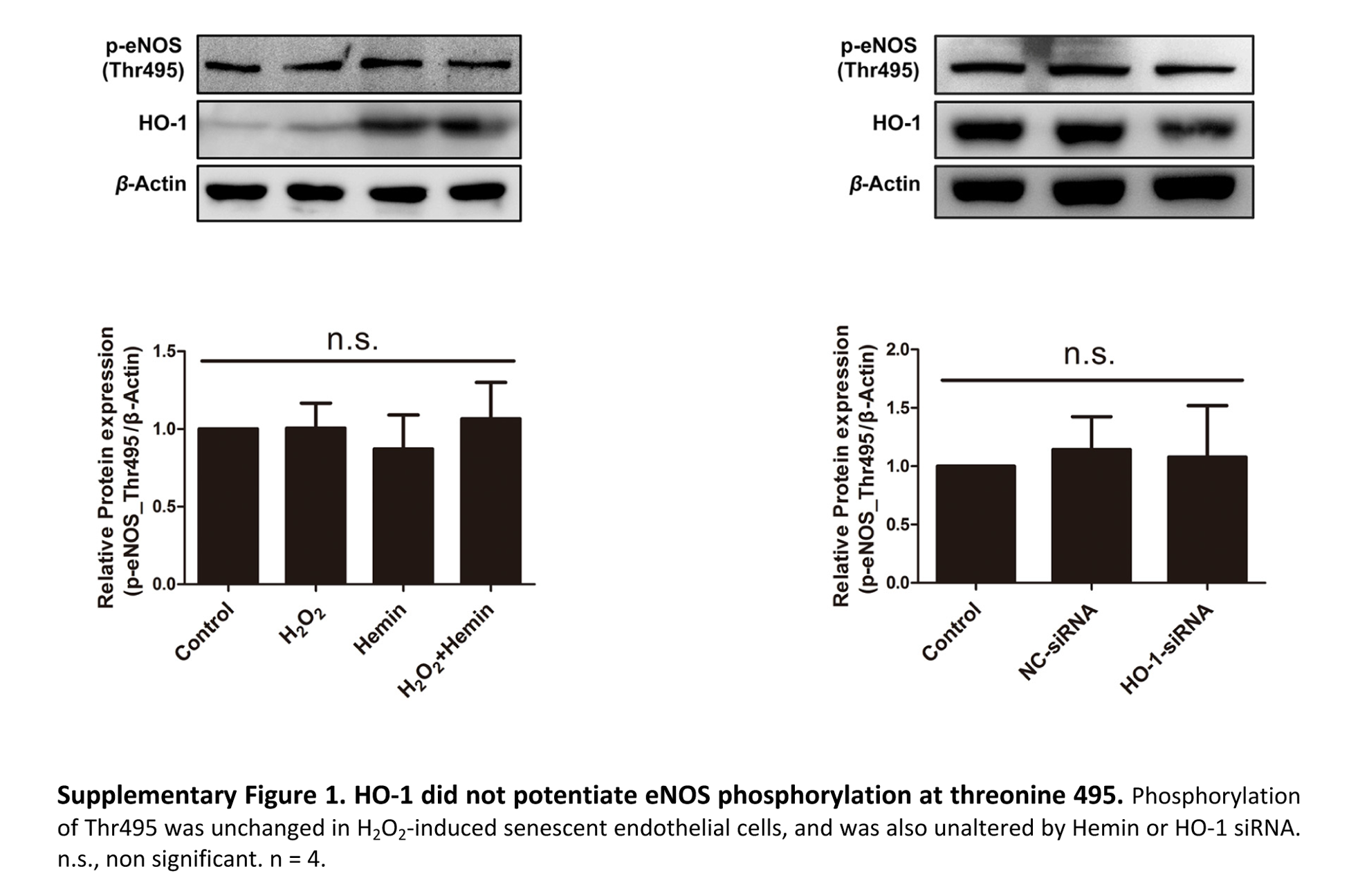

Supplement: Supplementary Figure 1 [file aging-10-101506-s001.tif]

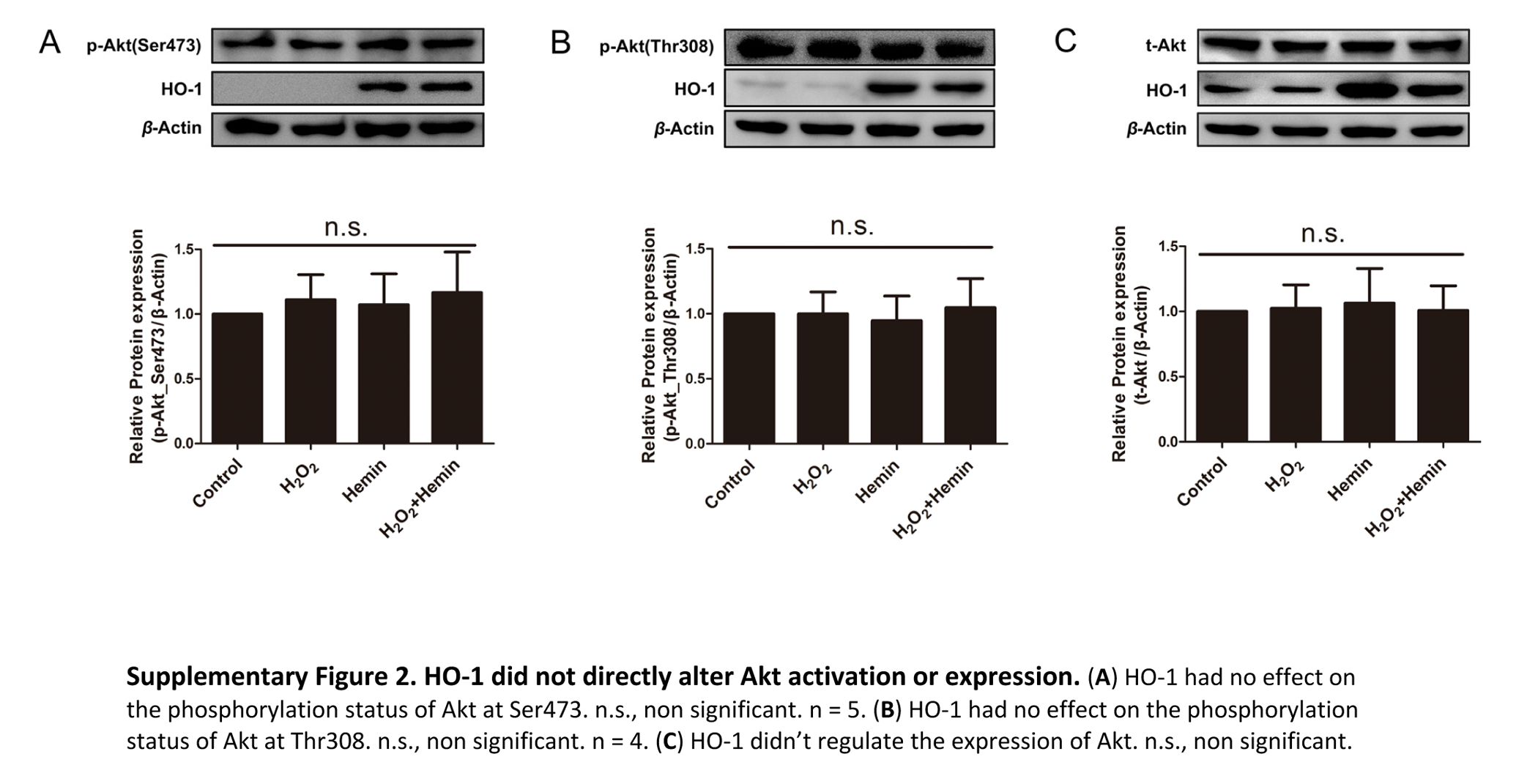

Supplement: Supplementary Figure 2 [file aging-10-101506-s002.tif]

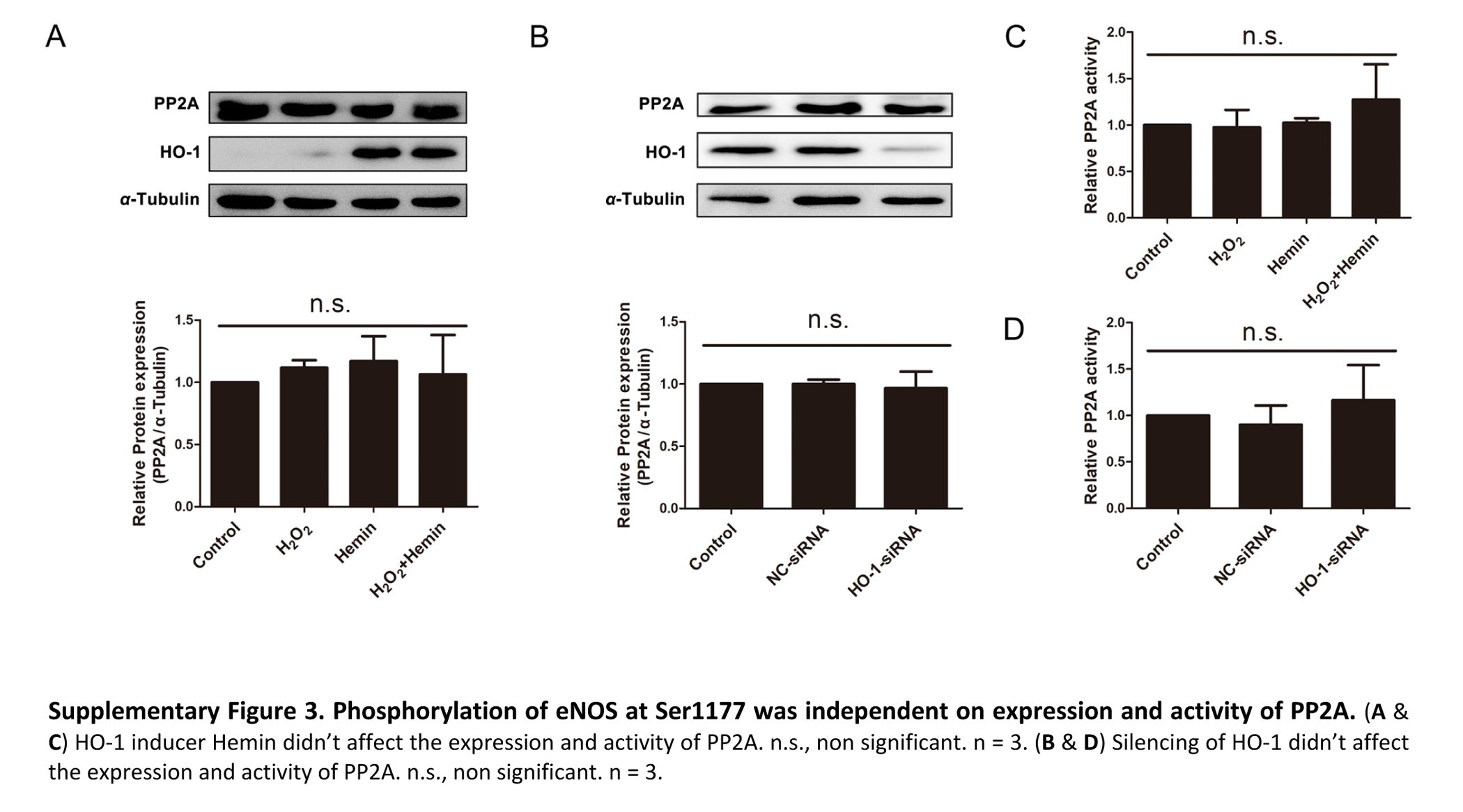

Supplement: Supplementary Figure 3 [file aging-10-101506-s003.tif]

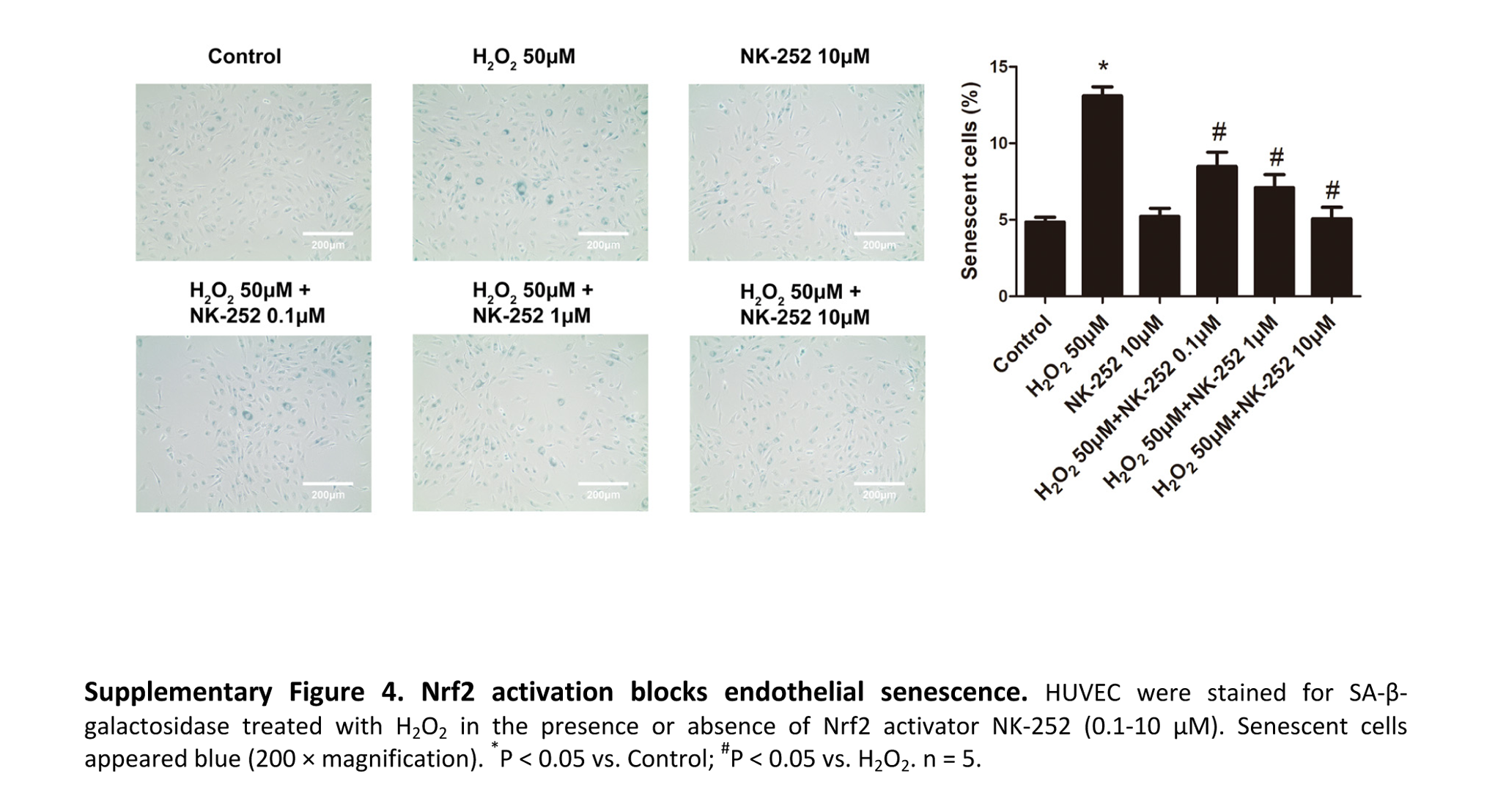

Supplement: Supplementary Figure 4 [file aging-10-101506-s004.tif]

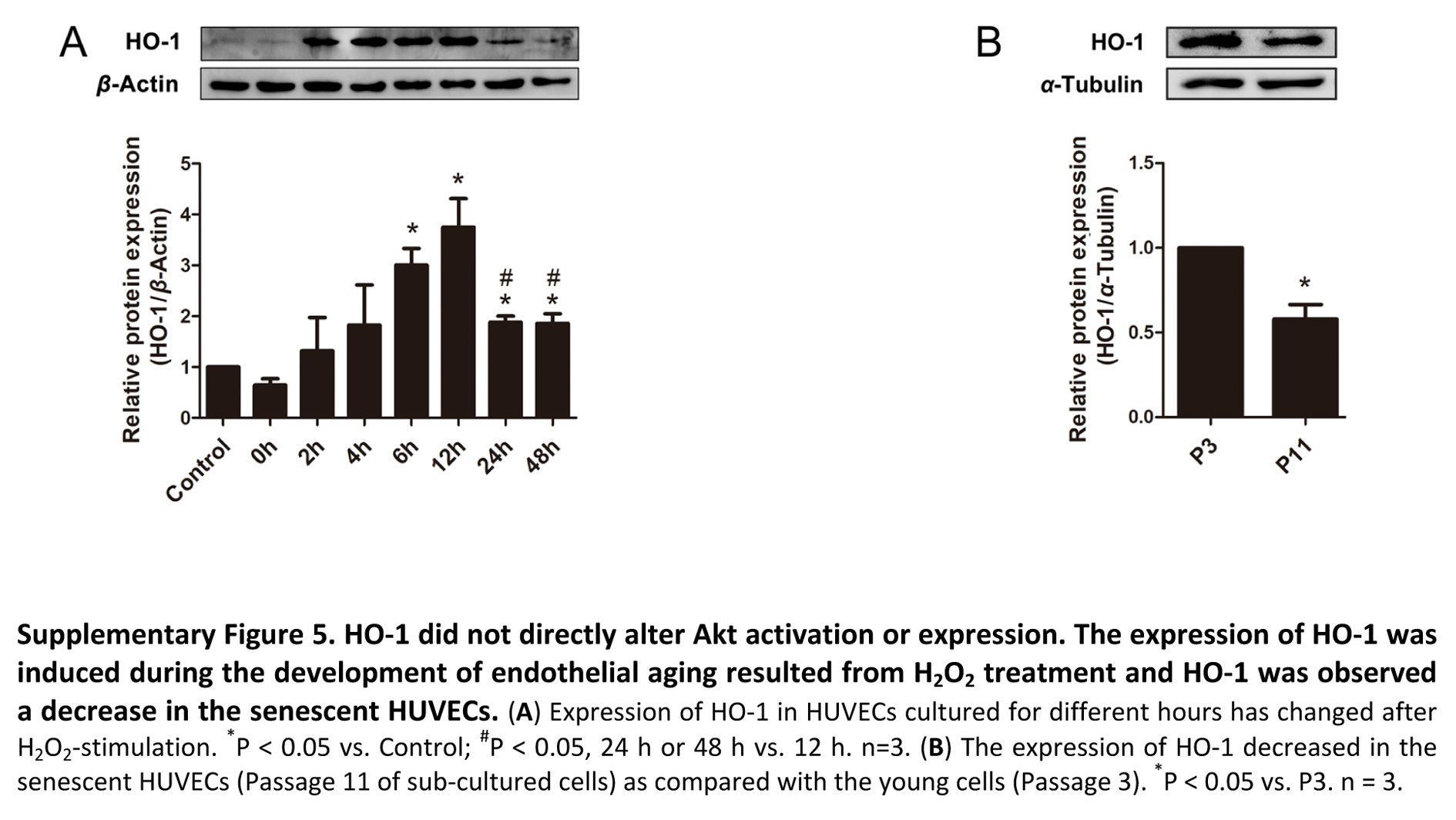

Supplement: Supplementary Figure 5 [file aging-10-101506-s005.tif]
